# Polycations as Aptamer-Binding Modulators for Sensitive Fluorescence Anisotropy Assay of Aflatoxin B1

**DOI:** 10.3390/s24103230

**Published:** 2024-05-19

**Authors:** Alexey V. Samokhvalov, Alena A. Mironova, Sergei A. Eremin, Anatoly V. Zherdev, Boris B. Dzantiev

**Affiliations:** 1 A.N. Bach Institute of Biochemistry, Research Center of Biotechnology, Russian Academy of Sciences, 119071 Moscow, Russia; 03alexeysamohvalov09@gmail.com (A.V.S.); mironova.alena2002@yandex.ru (A.A.M.); zherdev@inbi.ras.ru (A.V.Z.); 2 Faculty of Chemistry, M.V. Lomonosov Moscow State University, 119991 Moscow, Russia; saeremin@gmail.com

**Keywords:** stem–loop aptamer, mycotoxin, fluorescein, fluorescence polarization, polyethylene glycol, poly-L-lysine

## Abstract

Fluorescence induced by the excitation of a fluorophore with plane-polarized light has a different polarization depending on the size of the fluorophore-containing reagent and the rate of its rotation. Based on this effect, many analytical systems have been implemented in which an analyte contained in a sample and labeled with a fluorophore (usually fluorescein) competes to bind to antibodies. Replacing antibodies in such assays with aptamers, low-cost and stable oligonucleotide receptors, is complicated because binding a fluorophore to them causes a less significant change in the polarization of emissions. This work proposes and characterizes the compounds of the reaction medium that improve analyte binding and reduce the mobility of the aptamer–fluorophore complex, providing a higher analytical signal and a lower detection limit. This study was conducted on aflatoxin B1 (AFB1), a ubiquitous toxicant contaminating foods of plant origins. Eight aptamers specific to AFB1 with the same binding site and different regions stabilizing their structures were compared for affinity, based on which the aptamer with 38 nucleotides in length was selected. The polymers that interact reversibly with oligonucleotides, such as poly-L-lysine and polyethylene glycol, were tested. It was found that they provide the desired reduction in the depolarization of emitted light as well as high concentrations of magnesium cations. In the selected optimal medium, AFB1 detection reached a limit of 1 ng/mL, which was 12 times lower than in the tris buffer commonly used for anti-AFB1 aptamers. The assay time was 30 min. This method is suitable for controlling almond samples according to the maximum permissible levels of their contamination by AFB1. The proposed approach could be applied to improve other aptamer-based analytical systems.

## 1. Introduction

**Figure 1:**
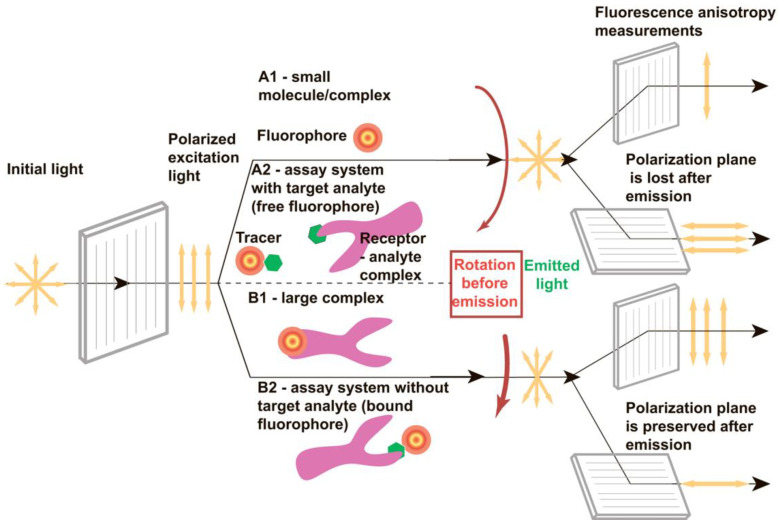
The influence of rotational motion on the anisotropy of fluorescence that is emitted after excitation by plane-polarized light. A1, A2—cases of rapid rotation, B1, B2—cases of slow rotation. Additional comments are given in the text.

Fluorescence anisotropy (FA) is an efficient parameter to characterize intermolecular interactions and to detect different compounds [1,2]. Excitation with plane-polarized light and recording the degree of polarization of the emitted light allows for registering interactions of fluorophore-containing reagents simply and rapidly. Figure 1 demonstrates these possibilities. Since fluorophore or the fluorophore-containing complex rotates in solutions chaotically due to Brownian motion, the polarization plane of the exciting light is not preserved during emission [3]. By recording emission in planes parallel and perpendicular to the excitation plane, it is possible to estimate the degree of depolarization. The more mobile the fluorophore-containing complex is in the solution, the less the emitting light retains the polarization of the exciting light (Figure 1, A1). If a small fluorophore binds to a large compound, the rotation of the complex slows down, and the polarization of the emitted light becomes high (Figure 1, B1). If some process prevents the fluorophore from binding to the receptor, the fluorophore remains in the solution as a small molecule, rotates rapidly, and emits depolarized light.

To quantitatively describe the depolarization of emitted light in such systems, fluorescence anisotropy is calculated as follows:

FA = (I_||_ − I_⊥_)/(I_||_ + 2I_⊥_) (1)

where I_||_ is the intensity of the emitted light in the plane parallel (||) to the direction of the polarized excitation, and I_⊥_ is the intensity of the emitted light in the perpendicular plane (⊥) [3].

Note that fluorescence polarization (FP) is often used as alternate parameter characterizing such systems.

FP = (I_||_ − I_⊥_)/(I_||_ + I_⊥_) (2)

However, the FA makes it easier to calculate the concentrations of the reacting molecules and their complexes [3].

The above-mentioned features of fluorescence anisotropy induced by plane-polarized light make the FA-based detection of various compounds of low molecular weights possible. The implementation of the FA assay consists of mixing (i) a sample potentially containing the target analyte with solutions of (ii) an analyte-specific receptor and (iii) the analyte modified with a fluorophore (so-called tracer). The free tracer rotates rapidly (Figure 1, A2), whereas its binding with the receptor slows down the rotation (Figure 1, B2). Therefore, as the analyte concentration in the sample increases, the proportion of the tracer bound to the receptor molecule decreases, and a lower value of fluorescence anisotropy is registered [4,5,6].

Many immunoanalytical systems based on this effect have been developed and successfully used to detect different compounds [1,7]. The key advantages of these techniques are homogeneous interactions in the solution, simple one-step implementation with real-time modes of detection, and limited influence of sample matrixes due to the use of a ratio between two registered intensities [3,8].

In recent years, aptamers have been used actively as alternative receptor molecules in various analytical systems. Aptamers are single-stranded oligonucleotides that can bind molecular targets with high affinity and selectively bind not inferior ones of antibodies [9,10]. Their advantages over antibodies are low-cost production via chemical synthesis, high stability, and simple modification [11,12]. However, due to the smaller size (10–20 kDa vs. 150 kDa for IgG) and intramolecular structural flexibility [4,13], the aptamer–tracer reaction leads to less changes in FA, limiting the possibilities of sensitive and accurate assays.

Therefore, along with traditional schemes identical to the competitive analysis using antibodies [7], alternative approaches with the amplification of the registered FA signals are actively developed [2,4]. The considered approaches include using labels that interact with nucleobases [14], ligand-induced strand displacement [15], and aptamer modification by proteins and nanoparticles as anchors slowing down their rotation [15,16]. However, the experience with alternative approaches is limited, and the degree of their versatility and effectiveness are unclear.

It is well known that a medium can influence the structure of oligonucleotides; this effect is actively used in DNA condensation protocols [17,18] but was not earlier considered in connection with FA-based assays. Such potential modulators of aptamer structures and mobility are polycations (poly-L-lysine, etc.), nonionic polymers (polyethylene glycol, etc.) [19,20,21], and divalent metal cations (Mg^2+^, Ca^2+^, Ba^2+^, Zn^2+^) [22,23]. Note that their use should integrate the stabilization of the aptamer structure and slow down its rotation while maintaining their analyte-binding properties.

Our study has been implemented using an interacting pair of aflatoxin B1 (AFB1) and an anti-AFB1 aptamer. AFB1 is a fungal metabolite, widespread toxic contaminant of foodstuffs with multiple hazardous effects on human health [24]. Among known AFB1-binding aptamers, the untruncated stem–loop oligonucleotide 5′-GTT GGG CAC GTG TTG TCT CTC TGT GTC TCG TGC CCT TCG CTA GGC CCA CA-3′ [25] was chosen as a high-affine reactant with a row of structurally studied modifications. Thus, the 3D structure of its 26-mer truncated variant in the complex with AFB1 was determined by NMR [26]. The previous developments of FA-based assays of AFB1 include immunoassays [27,28,29], whereas, in the case of aptamers, only different complicated variants with aptamer labeling were realized [14,15,30,31]. The competitive format of FA-based aptamer assay for AFB1 with analyte labeling has not previously been considered.

This study aimed to develop a novel strategy for increasing the sensitivity of FA aptamer-based assays via the use of compounds capable of weak interactions with the aptamer. We investigated the uses of poly-L-lysine (PLL), polyethylene glycol (PEG), and Mg^2+^ ions. The assessment of efficiency for the developed assay included its use for AFB1 detection in almond samples and comparison with the existing analytical techniques.

## 2. Materials and Methods

### 2.1. Reagents and Sample Preparation

The AFB1 powder was obtained from Sigma-Aldrich (St. Louis, MO, USA), and its standard solution for HPLC in acetonitrile (10 μg/mL) from Trilogy (Austin, TX, USA). The DNA aptamers (see Table 1) and their 5′-biotinylated derivatives were custom-synthesized and purified by Syntol (Moscow, Russia). Tris(hydroxymethyl)aminomethane, dimethyl sulfoxide (DMSO, 99.9%), sodium acetate, poly-L-lysine (30–70 kDa), and polyethylene glycol (8 kDa) were from Sigma-Aldrich (USA); recombinant streptavidin was obtained from IMTEK (Moscow, Russia); and magnesium acetate, methanol (MetOH) (99.8%), glycerol (99.8%), and Tween-20 were from Honeywell (Charlotte, NC, USA). All chemicals were analytical grade or chemical reagent grade.

A Simplicity Milli-Q^®^ system from Millipore (Darmstadt, Germany) was used to obtain ultrapure water for buffers and reagent solutions. Stock solutions of aptamers were prepared by dissolving lyophilized DNA in deionized water that had been filtered through a 0.22 μm membrane. A NanoDrop2000 microvolume spectrophotometer (Thermo Scientific, Waltham, MA, USA) was used to measure the concentrations of aptamers by optical density at 260 nm.

All fluorescence anisotropy (FA) measurements were performed in black non-binding 96-well microplates from Thermo Scientific NUNC^TM^ (Roskilde, Denmark), using a CLARIOstar multimode plate reader (BMG Labtech, Ortenberg, Germany). The excitation filter was 490 ± 10 nm, the dichroic mirror was 504 nm, and the emission filter was 520 ± 10 nm.

### 2.2. Synthesis of the Fluorescein-Labeled Derivative of Aflatoxin B1 (AFB1-EDF)

Aflatoxin B1-1-(O-carboxymethyl)oxime was synthesized from AFB1 and carboxymethoxylamine as described previously [28]. Fluoresceinthiocarbamylethylenediamine (EDF) was synthesized by isothiocyanate coupling FITC with one of the primary amine groups of ethylenediamine. The carbodiimide protocol [28] with modifications was used for the synthesis of AFB1, labeled with fluoresceinthiocarbamylethylenediamine via a carboxymethoxylamine linker (AFB1-EDF). In total, 3.6 mg of AFB1 oxime was dissolved in 0.2 mL of dimethylformamide, which contained 3 mg of N-hydroxysuccinimide and 5 mg of N′-dicyclohexylcarbodiimide, and, after stirring, was incubated overnight at room temperature. Then, 0.8 mg of EDF was added. The mixture was incubated in the dark for 24 h; then, a small portion (about 10 μL) was purified by thin-layer chromatography with the chloroform: methanol eluent (4:1 *v*/*v*). The stock AFB1-EDF solution was prepared by dissolving the main band (Rf = 0.7) in methanol and was stored at 4 °C.

### 2.3. The Characterization of the Interactions between Aptamer Variants and AFB1-EDF Using Fluorescence Anisotropy

A series of 3-fold consecutive dilutions of anti-AFB1 aptamers from 20 to 0.0006 μM were prepared in a 96-well microplate. To each well that contains 50 μL of an aptamer, 10 nM of AFB1-EDF was added to the final volume of 100 μL in tris buffer (TB—20 mM Tris-Acetate, 100 mM NaAcetate, and 20 mM MgAcetate_2_, pH 8.4). Wells that contain TB instead of aptamer were used as negative controls. The microplate was gently shaken for two min at 25 °C, after which the fluorescence intensity in reference fluorescence units (RFU) and FA values in milliunits were measured. The data were analyzed using CLARIOstar MARS software v4.00 R2. The ∆FA values were calculated as the difference between FA values of AFB1-EDF with and without aptamer.

### 2.4. The Determination of Dissociating Constants Using Fluorescence Anisotropy Measurements

The dissociation constant (K_D_) of aptamer interactions with AFB1-EDF was calculated as described in [32]. Briefly, the dependencies of FA from aptamer concentration were approximated by a four-parameter sigmoid fitting with the use of Origin 8.1 software (Origin Lab, Northampton, MA, USA).

(3) Y=Y0+Ymax−Y01+(xx0)p

where *X* is the concentration of the aptamer; *Y* is the FA; *Y_max_* is the FA for an infinitely high concentration of the aptamer (upper asymptote); *Y*_0_ is the FA for an infinitely low concentration of the aptamer (lower asymptote) in milliunits of anisotropy; *X*_0_ is the concentration of the aptamer at the inflection point (the point of 50% binding); and *p* is the slope of the curve at the inflection point.

Then, the part of the bound labeled ligand (*F_bound_*) was calculated using the following equation:

(4) $$F_{bound}=\frac{Y_{i}-Y_{0}}{Q(Y_{max}-Y_{i})+Y_{i}-Y_{0}}$$

where *Q* is a correction factor calculated as the proportion between fluorescence intensities of the bound and unbound label.

To obtain the dissociation constants of the AFB1-EDF interaction with aptamers the experiments were carried out at AFB1-EDF concentration smaller by an order of magnitude than K_D’_s [AFB1-EDF*]/K_D_ < 0.1. Under this condition, the K_D_ is equal to the aptamer concentration at the point of 50% binding in the half-logarithmic coordinates of *F_bound_* from the aptamer concentration.

### 2.5. The Characterization of Influence of Different Medium Compounds or Co-Solvents on the Fluorescence Anisotropy

Consecutive dilutions of medium compounds, co-solvents, and their mixtures were prepared in deionized water in a 96-well microplate. Then, to 50 μL of each dilution, equal volumes of aptamer 38 nt and AFB1-EDF in 2× solution of studied buffer were added, providing their final concentrations of 100 and 5 nM, respectively. To measure the FA of unbound AFB1-EDF, the aptamer solution was replaced with the same volume of 2× buffer. The final volume in each well was 100 µL. Then, the microplate was gently shaken for 2 min, and the fluorescence and anisotropy of fluorescence were measured.

The following compounds were investigated in TB:

(1) The 5-fold dilutions from 10 to 0.08 μM of poly-L-lysine, 30 kDa;
(2) The 5-fold dilutions from 8750 to 70 μM of polyethylene glycol, 8 kDa;
(3) The 3-fold dilutions of MgAcetate_2_ from 1 M to 37 mM in a magnesium-free TB.

The following co-solvents in the range of concentration from 50 to 1.8% (*v*/*v*) were studied: (1) DMSO; (2) glycerol; and (3) MetOH.

The following buffer variants in magnesium-free TB were studied:

#1—1 M MgAcetate_2_ and 0.4 μM PLL;

#2—1 M MgAcetate_2_ and 70 μM PEG;

#3—1 M MgAcetate_2_, 0.4 μM PLL, and 70 μM PEG;

#4—0.3 M MgAcetate_2_ and 0.4 μM PLL;

#5—0.3 M MgAcetate_2_ and 70 μM PEG;

#6—0.3 M MgAcetate_2_, 0.4 μM PLL, and 70 μM PEG.

### 2.6. The FA-Based Detection of Aflatoxin B1

Competitive interactions between AFB1 and AFB1-EDF for binding to the aptamer were carried out in TB and buffer variant #6 with 0.1% Tween20. The ten successive 3-fold dilutions of AFB1 were prepared in deionized water containing 6% (*v*/*v*) MetOH. Then, to 50 µL of each dilution, the equal volumes of aptamer 38 nt and AFB1-EDF in the 2-fold buffer were added to a final concentration of 100 (in TB) or 25 nM (in buffer #6) and 5 nM, respectively. Wells containing AFB1-EDF mixed with deionized water and buffer or with deionized water and aptamer were used as positive and negative controls, respectively. The linear range of the fitting curve in half-logarithmic coordinates was calculated in accordance with [33]. The limit of detection was calculated as the concentration corresponding to the triple standard deviation from the mean value of the blank sample.

### 2.7. Sample Preparation

Almond flour was bought from a local convenience store. To evaluate AFB1 recovery, spiked samples of almond flour with known AFB1 concentration were prepared. Almond flour was extracted using a mixture of methanol and water at a ratio of 70:30 [34]. The resulting mixture was centrifuged for 30 min at 6500× *g* using AmiconUltra-15 centrifugal filter units (Millipore, Burlington, MA, USA) with molecular weight cut-off 3 kDa. The AFB1 measurements were carried out in 10-fold diluted extracts.

Eight almond samples were tested in the V.M. Gorbatov Federal Research Center for Food Systems of the Russian Academy of Sciences (Moscow, Russia). The extraction of AFB1 from 10 g of each almond flour sample was carried out using 20 mL of methanol: water mixture (70:30 *v*/*v*). The AFB1 content was measured by HPLC-MS/MS as described in [35].

## 3. Results and Discussion

### 3.1. The Scheme of the Proposed Fluorescence Anisotropy Assay for Aflatoxin B1 Using Polycation Modulators

**Figure 2:**
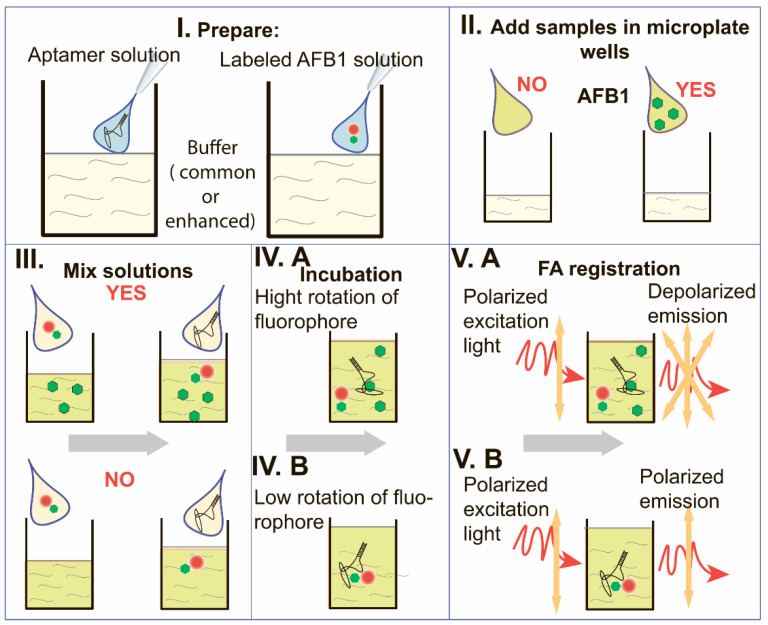
Flow chart of the proposed polycation-mediated fluorescence anisotropy assay. (**I**). Preparation of aptamer solution and AFB1-fluorophore conjugate solution. (**II**). Addition of samples without or with AFB1 to microplate wells. (**III**). Addition of the solutions prepared at the step 1 to the wells. (**IV**). Incubation of the reaction mixture ((**A**)—the case with AFB1 in the sample, (**B**)—the case without AFB1 in the sample). (**V**). Registration of FA in the reaction mixture ((**A**)—the case with AFB1 in the sample, (**B**)—the case without AFB1 in the sample).

The proposed assay technique is very simple in implementation and consists only of mixing the necessary reagents, their incubation, and the following FA measurements directly in the reaction mixture without any separating or enhancing stages. Figure 2 illustrates the assay principle.

The reaction media containing buffer compounds, polycations, and magnesium salt was prepared. Stock solutions of the aptamer specific to AFB1 and the tracer (AFB1-fluorophore conjugate) were added to the reaction media (step I). Samples to be tested (including AFB1-negative and AFB1-positive ones) were added to the wells of the black microplate for fluorescent measurements (step II). Then, the prepared solutions labeled AFB1 and aptamer were added to the samples (step III). Incubation at room temperature provides the possibility for competitive interaction with the aptamer (step IV.A/IV.B). The content of AFB1 in the sample determines the proportion of free and bound states of the tracer and, in this way, the anisotropy of the emitted light (step V.A/V.B). The more AFB1 in the sample, the fewer tracers bind to the aptamer and the lower the FA value.

Note that based on the theory of fluorescence polarization, the FA values are determined by factors such as the half-life time of excited fluorophore, coefficient of rotational diffusion, size and shape of the rotating compounds, temperature, and viscosity of the solvent [3,4]. Therefore, the modulation of these factors could influence the parameters of FA-based assays.

### 3.2. The Influence of the Length of the Aptamer Stem Region on the Binding of Labeled AFB1

We characterized the influences of pH and salt composition on the dissociation constant of the complex between the untruncated aptamer and AFB1-EDF using FA measurements, as shown in Figures S1 and S2. The chosen buffer is consistent with previous studies [14,26,31] and consists of 20 mM Tris-Acetate, 100 mM NaAcetate, and 20 mM MgAcetate_2_, pH 8.4 (TB).

### 3.3. The Influence of the Length of the Aptamer Stem Region on the Binding of Labeled AFB1

**Figure 3:**
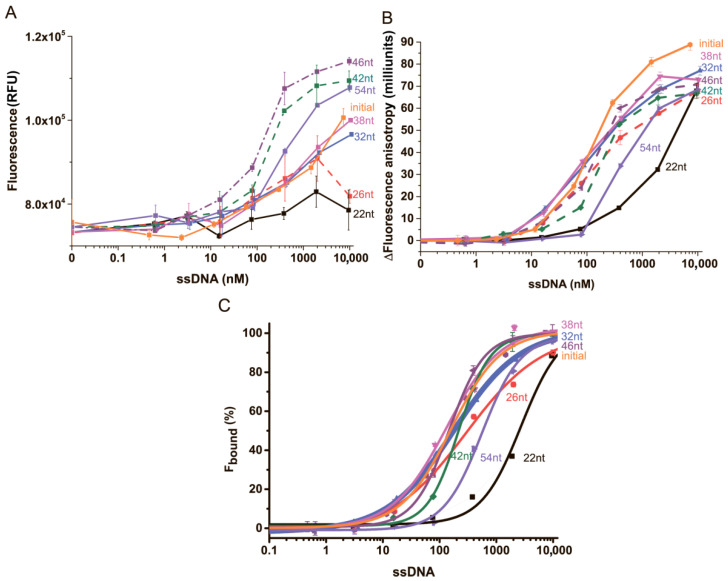
(**A**) The dependencies of fluorescence (**A**), the change in fluorescence anisotropy (**B**), and the percentage of bound fraction (**C**) of AFB1-EDF on the concentration of (orange) initial aptamer; (black) aptamer 22 nt; (dash, red) aptamer 26 nt; (blue) aptamer 32 nt; (magenda) aptamer 38 nt; (dash, olive) aptamer 42 nt; (dash, purple) aptamer 46 nt; (dash, violet) aptamer 54 nt (*n* = 3).

Seven truncated variants of the aptamer with stem lengths ranging from 4 to 18 and an initial aptamer were compared by their interaction with AFB1-EDF. The values of total fluorescence and FA dependences on anti-AFB1 aptamer concentrations were measured. The FA data were processed and presented as the difference between the maximum FA value when the label was completely bound to the aptamer and the minimum FA in the absence of an aptamer (∆FA = FA_aptamer-AFB1-EDF_ − FA_AFB1-EDF_) to compare curves for different aptamers. Then, the fraction of bound AFB1-EDF was calculated from FA data and used to calculate K_D_ of AFB1-EDF complex with aptamers, as elucidated in Section 2.5. All data are summarized in Figure 3 and Table 1.

As we can see, the increase in the length of the stem region (see lines from 2 to 7 in Table 1) of the aptamer is accompanied by an increase in label fluorescence (up to 1.6-fold for the 46 nt variant with stem consisting of 14 nucleotide base pairs (bp)), as shown in Figure 3A. The exception is the 54 nt variant. In the case of FA, as can be seen in Figure 3B, a broad optimum is observed in the range of lengths of stem region between 4 and 14 bp (see lines from 3 to 7 in Table 1). In the studied conditions, the variant with a stem of 2 bp (line 2) retains AFB1-binding activity, although with a significantly worse K_D_ value (by order of magnitude) than the initial aptamer. The increase in the length of the stem seems to be limited by the self-annealing of the stem–loop aptamer [36]. We hypothesized that this factor caused the low values of fluorescence and FA that we obtained for the aptamer 54 nt in TB.

To date, several truncated variants of the aptamer have been proposed for application in assays. The 26-nucleotide variant has a minimal length that provides the affinity of aptamer. Below this length, the reduction in binding abilities is observed [14,25,26,37]. The 3D structure of the 26-nucleotide variant was previously determined [26] and showed that AFB1 binds in the 18-nucleotide-long loop region. The stem region does not directly interact with AFB1 but is necessary for stabilizing the loop.

Based on these data, we decided to provide an additional stabilization of the loop by the elongation of the stem region, as well as to compare the obtained variants with previously proposed 26 nt, of which its parameters are presented in line 3 in Table 1. The given three aptamer variants with an elongated stem region of 10 to 14 bp (duplex consisting of from 11 up to 37 hydrogen bonds), lines 5–7 in Table 1, showed dissociation constants and FA values for the complex that were close to those of the initial aptamer and minimal aptamer. The aptamer variant 38 nt was chosen for further study.

### 3.4. The Influence of Polymers and Cations on Fluorescence Anisotropy of Fluorescein-Labeled AFB1 in Free and Bound State

**Figure 4:**
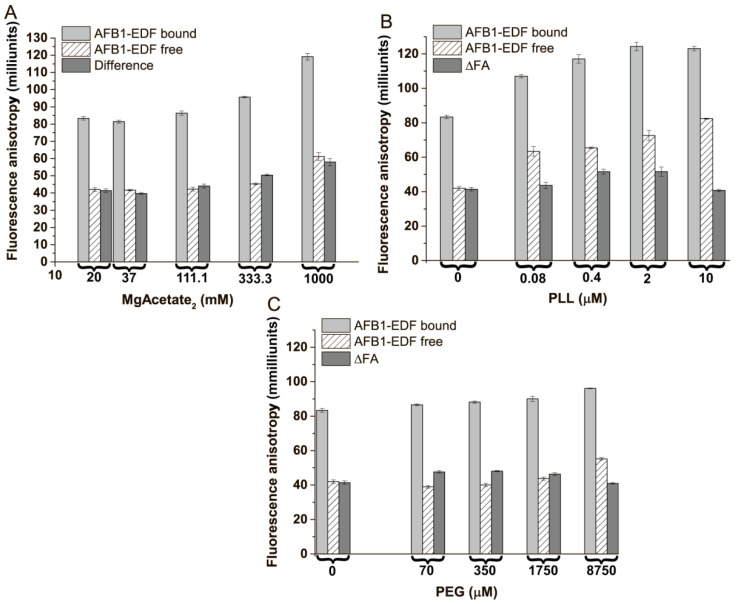
The fluorescence anisotropy of AFB1-EDF in the absence and presence of 100 nM of aptamer 38 nt and the ΔFA in: (**A**) – 20 mM Tris-Acetate, 100 mM NaAcetate, ph 8.4, with different MgAcetate2 concentrations and TB with different PLL (**B**) and PEG (**C**) concentrations (*n* = 3).

We implemented several strategies to change the FA response during aptamer 38nt–AFB1 complexation. The first strategy involves modifying the state of a DNA-based aptamer using compounds that weakly interact with the DNA, like polycations and some neutral polymers. The idea of polycationic additions is based on a decrease in the repulsion between DNA segments that led to the compacting of a DNA structure [38]. Also, a decrease in repulsion between a DNA and a fluorescein is occurring, restricting the segmental and local motion of the label [4]. This effect was associated with the depolarization of fluorescein fluorescence in oligonucleotide complexes [4]. The increase in fluorescein FA in its covalent complexes with oligonucleotides is observed after the addition of poly-L-lysine [20] and divalent cations [22]. Also, several studies indicate that Mg^2+^ (in concentrations up to 0.1 M) plays a crucial role in the affinity of AFB1 binding by the aptamer [14,25]. It is known that PEG is commonly used with polycations for DNA condensation [17,19,39] and stabilization [40] and can weakly interact with it [19]. However, its applicability in increasing the ∆FA during aptamer–ligand complexation has not been previously demonstrated. We chose these compounds for further investigation. The concentration of aptamer 38 nt at 100 nM, close to the IC50 value obtained in Section 3.2, was chosen for the complexation of AFB1-EDF with the aptamer. The obtained data on the FA of AFB1-EDF at the free and bound states and ∆FA at various concentrations of Mg^2+^, PLL, and PEG are presented in Figure 4.

Figure 4A shows that, in the presence of Mg^2+^, the FA steadily increases for the AFB1-EDF complex with the aptamer 38 nt. The stem–loop aptamer maintains its ligand-binding ability up to a magnesium cation concentration of 1 M, and the total ΔFA increase as compared with the initial buffer is 16 milliunits of anisotropy (or 38%). In the case of PLL, the FA of the complex reaches a maximum of 2 μM. The maximum ΔFA of 10 milliunits (or 18%) was achieved at 0.4 mM PLL. The results demonstrate that PLL and magnesium increase the FA response of the fluorescein label that is complexed non-covalently with the aptamer. On the other hand, the increase in FA of free AFB1-EDF in the presence of PLL or at magnesium concentrations above 300 mM indicates the presence of AFB1-EDF interaction with high concentrations of polycations. In the case of PEG, only a little increase in FA of both the bound and free state of AFB1-EDF was observed, leading to a maximum ΔFA gain of 7 milliunits (or 9%) at a concentration of 70 μM.

### 3.5. The Influence of Different Co-Solvents on Fluorescence Anisotropy of Fluorescein-Labeled AFB1 in the Free and Bound State

**Figure 5:**
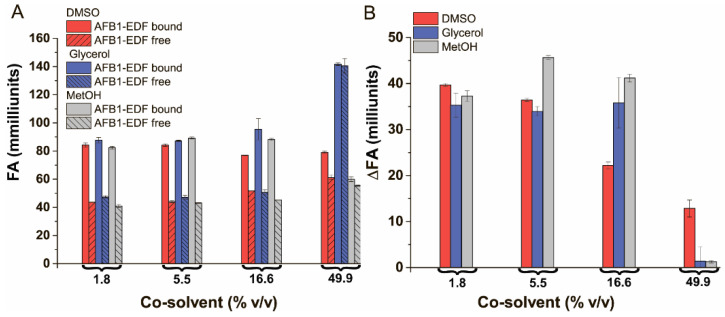
The FA (**A**) and the ∆FA (**B**) of AFB1-EDF in the absence and in the presence of 100 nM of the aptamer 38 nt in TB, contain different % (*v*/*v*) of organic solvents (*n* = 3).

The addition of different co-solvents was investigated next. DMSO is known for its ability to reduce the persistence length of DNA and can distort DNA duplexes [41,42] by forming both the strong hydrophobic interactions [42] and hydrogen bonds [43] between DNA and DMSO moieties. Glycerol is known for its high viscosity and the ability to preserve the DNA duplex structure [41]. MetOH can induce DNA condensation in the presence of divalent metal cations [44] and is commonly used as an extractant to prepare food extracts. The obtained data on the FA of AFB1-EDF at free and bound states and ∆FA at various concentrations of DMSO, glycerol, and methanol are shown in Figure 5.

The data show that DMSO interferes with the aptamer–AFB1-EDF interaction in concentrations above 10% (*v*/*v*), as indicated by the anisotropy measurements. An increased FA in the free AFB1-EDF state and a decreased FA in its bound state have been observed. Glycerol and methanol show little effect on FA at concentrations below 16.5% *v*/*v*. At 50% (*v*/*v*), all three co-solvents studied led to a decrease in ΔFA between the bound and free states of AFB1-EDF. Thus, none of the studied co-solvents have shown the desired effect, except methanol to a certain degree. However, a small amount of it is already present in real samples as an extractant.

### 3.6. The Influence of the Aptamer Complexation with Streptavidin on Fluorescence Anisotropy

The second strategy was based on the increase in the aptamer size by its inclusion in the complex with streptavidin (anchor) via biotin attached to 5′-end. In the case of the solid sphere model, the FA is directly proportional to the size of the fluorescent molecule [3]. Similar dependencies, as in the previous experiments of FA and ∆FA, were obtained, as shown in Figure S3. In this case, the attachment of 65 kDa streptavidin to the end of the aptamer 38 nt stem region did not have any effect on the FA of AFB1-EDF. This implies the independence of the AFB1-EDF motion from the stem region of the stem–loop aptamer and restricts the application of this strategy previously confirmed for the anti-ochratoxin A aptamer forming an antiparallel G-quadruplex [16]. Note that the strategy was efficient, as shown in the systems based on a similar anti-AFB1 aptamer when the label is introduced in the aptamer, and the anchor is introduced in the complementary strand [15,30].

### 3.7. The Cooperative Influence of Polycations and PEG on the Difference in FA between the Bound and Free States of Labeled AFB1

**Figure 6:**
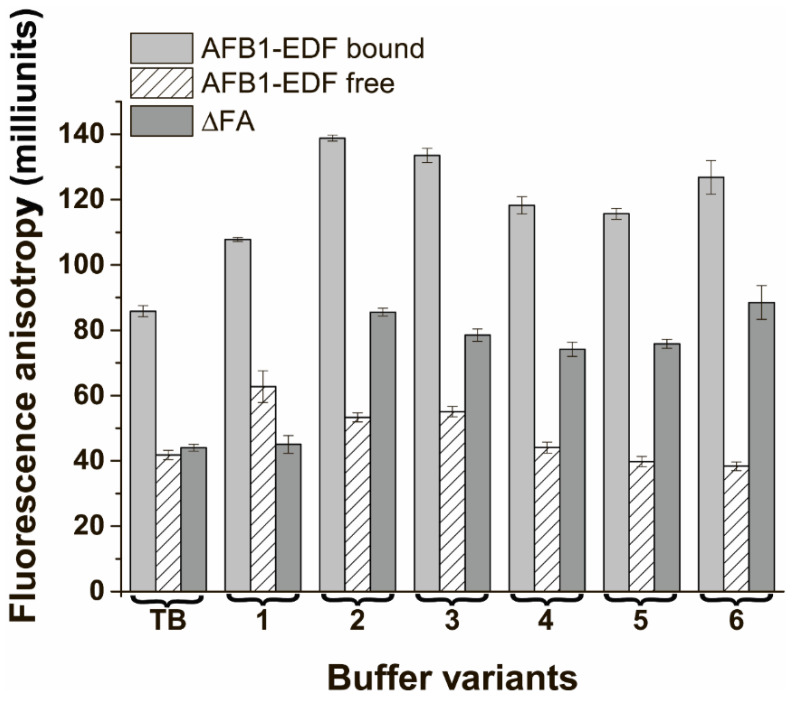
The fluorescence anisotropy of AFB1-EDF in the absence and presence of 100 nM of the aptamer 38 nt and ∆FA in TB and buffers based on 20 mM Tris-acetate, 100 NaAcetate, pH 8.4 with addition of #1—1 M MgAcetate_2_, 0.4 μM PLL; #2—1 M MgAcetate_2_, 70 μM PEG; #3—1 M MgAcetate_2_, 70 μM PEG, 0.4 μM PLL; #4—0.3 M MgAcetate_2_, 0.4 μM PLL; #5—0.3 M MgAcetate_2_, 70 μM PEG; #6—0.3 M MgAcetate_2_, 70 μM PEG, 0.4 μM PLL (*n* = 3).

To check more complicated effects on the FA of the bound and free AFB1-EDF, we have studied the mixtures of magnesium, PLL, and PEG. Based on the results obtained in Section 3.3, the concentrations of PLL and PEG were selected as 0.4 and 70 μM, respectively. The concentrations of magnesium of 1 M and 0.3 M have been chosen for further investigation. Therefore, six buffer mixtures were prepared, and the FA of AFB1-EDF and its complex with the aptamer 38 nt were determined, as well as the FA difference between them. The results are shown in Figure 6.

The mixing of the chosen compounds led to the achievement of a 2-fold increase in the FA difference between the bound and free state of the label in cases of buffer variants #2 and #6, which contain 1 M Mg^2+^ and 70 μM PEG, or 0.3 M Mg^2+^ and 0.4 μM PLL, and 70 μM PEG, respectively. The results indicate that the concentrations of PLL and magnesium separately and in the mixture should not exceed a certain level, above which they change the FA of the labeled AFB1, as shown in Figure 6 for buffers #1 and 4. Interestingly, the PEG addition prevents the interactions of the polycations with free AFB1-EDF. The combination of PEG with magnesium and PLL shows a cooperative effect for the modulation of ∆FA. Therefore, their mixtures can be used to increase the sensitivity of the competitive system. Buffers #2 and #6, shows the largest ∆FA values between the free and bound state of AFB1-EDF and were selected for further experiment.

### 3.8. The Characterization of FA of the Bound and Free State of AFB1-EDF at Different Concentrations of the Aptamer in the Selected Media

**Figure 7:**
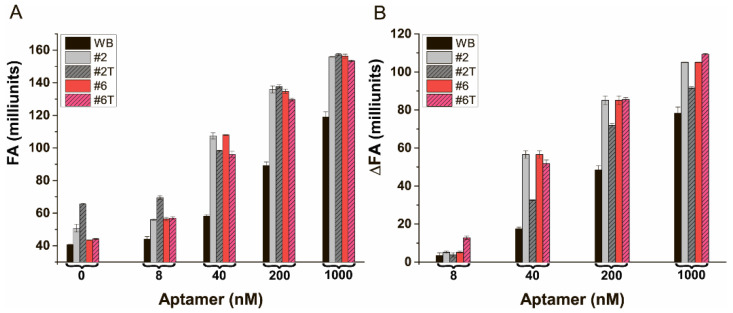
The fluorescence anisotropy (**A**) and fluorescence anisotropy changes (**B**) in AFB1-EDF under complexation with the aptamer 38 nt in TB and buffers: #2; #2T—same with 0.1% Tween20; #6; and #6T—same with 0.1% Tween20 (*n* = 3).

The next step was the assessment of FA dependencies of AFB1-EDF from aptamer 38 nt concentration in buffer variants #2 and #6 in comparison with TB. The FA and ∆FA dependencies are shown in Figure 7.

The complexation of AFB1-EDF with the aptamer 38 nt showed a better FA response in all designed media than in TB, see Figure 7A. The ∆FA values for the saturation of aptamer concentration when all AFB1-EDF is present in the bound state are greater by up to 40% in both the #2 and #6 medium compared to those in TB, see Figure 7B. Also, the ∆FA significantly increases at low concentrations of the aptamer. The obtaining increase allows the achievement of the same FA value with a lower concentration of the aptamer and shifts the parameters of competitive interaction to detect lower concentrations of AFB1. Additionally, the anisotropy values were investigated in these buffers containing 0.1% Tween20, see #2T and #6T in Figure 7. Tween20 is commonly used for reducing unspecific interactions [45]. As can be seen in Figure 7, the addition of Tween20 to medium #2 containing 1 M of magnesium led to a decrease in ∆FA. It indicated the occurrence of some interference during complexation, which was not observed under lower magnesium concentrations, see buffer #6. The buffer #6T was chosen for further approbation. To compare the FA competitive system in TB and medium #6T, the aptamer concentrations of 100 and 25 nM, providing the same ∆FA signal (about 45 units), were chosen, respectively.

### 3.9. The Comparison of FA-Based Aptamer Assays of AFB1 in TB and Buffer #6

**Figure 8:**
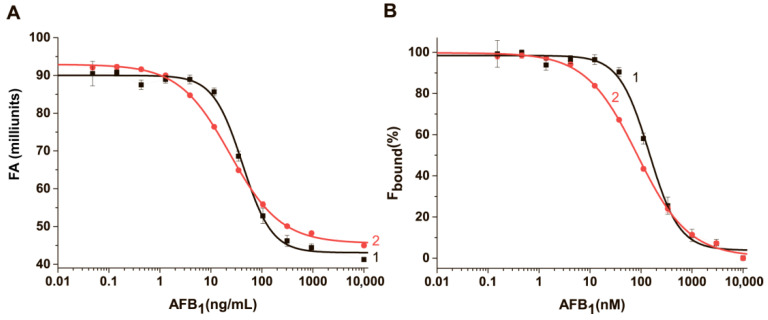
The dependencies of the fluorescence anisotropy (**A**) and percentage of bound (**B**) AFB1-EDF on the concentration of AFB1 in buffer (1) TB and (2) buffer #6T (*n* = 3).

The dependence of the FA of AFB1-EDF on the AFB1 concentration in TB and buffer#6T after they reached the equilibrium conditions was obtained, as shown in Figure 8A, and then processed to F_bound_ values, as shown in Figure 8B.

The linearity was within a concentration range of 4.0 ± 0.4–137.8 ± 8.0 ng/mL with an IC_50_ value of 23.4 ± 1 ng/mL in the buffer #6T. The linearity and IC_50_ values were 15.0 ± 3.4–117.4 ± 11.2 ng/mL and 41.6 ± 3.3 ng/mL in TB, respectively. The assay time was 30 and 15 min, respectively. The limit of detection (LOD) was 0.72 ± 0.3 and 9.7 ± 1.6 ng/mL in the #6T and TB, respectively. Using the mixture of cations (Mg^2+^ and PLL) and PEG instead of a simple buffer containing mono-and divalent cations allows the achievement of a 12-fold increase in sensitivity. The achieved LOD makes the developed system a promising tool for detecting AFB1 at the MRL level in nuts [46].

### 3.10. Detection of AFB1 in Food Samples

Nuts, such as almonds and pistachios, are susceptible to the presence of AFB1 [47], and the European Commission has established certain limits for the amount of AFB1 residues that are considered permissible in these products. In order to assess the recovery of AFB1, a series of spiked samples was prepared and then diluted with buffer #6T. The final dilution after mixing with analytical reagents was 10-fold. The concentration of methanol after dilution was 7% *v*/*v*. The resulting calibration curve in buffer #6T was used for sample analysis. As shown in Table 2, after a 60 min incubation, the recovery of AFB1 from almonds samples treated with 10, 30, 75, 100, and 200 μg/kg of AFB1 was within the range of 112–123%. The LOD and recovery values for the proposed FA-based competitive aptamer assay meet the EU criteria: the MRL of AFB1 is equal to 8–12 μg/kg in nuts [48].

The proposed technique was compared with HPLC-MS/MS in testing almond samples. The samples were 10-fold diluted, and the unified methanol content in the tested samples and the calibrating solutions was 14%. The results obtained by the two techniques demonstrate good accordance (see Table 3).

### 3.11. Comparison of the Developed FA Aptamer Assay with Other Methods

To date, several FA aptamer assays have been proposed for the detection of AFB1 based on the following: energy transfer effects during fluorescein-labeled aptamer absorption on graphene oxide [49], local effects of tetramethylrhodamine dye attached to a DNA molecule [14], ligand-induced strand displacement [15,30,50], and the combination of the ligand-induced stand displacement and the use of tetramethylrhodamine dye [31]. The use of polymerase chain reaction amplification and anchor-based methods makes the assay process more complex. However, it allows for the attainment of picomolar sensitivity. The analytical characteristics of the developed FA aptamer assays and those of the previously proposed homogeneous FA assays for the detection of AFB1 are compared and summarized in Table 4.

The described medium-assisted FA assay allows for a simple one-step detection of AFB1 in complex samples with comparable sensitivity to previously proposed systems. In terms of sensitivity, we are only inferior to complex multi-step approaches, making it a promising alternative for high-throughput measurements. The proposed novel approach indicates the perspective of the uses of more complex media, which contains positively charged and neutral polymers and high divalent ion concentrations for aptamer-based assays.

Considering the perspectives of the proposed approach and the further tasks required of its elaboration, the following limitations should be taken into consideration. First of all, as the proposed assay is a homogeneous one, the measurements are implemented directly in a reaction mixture without separation of its components. So, some matrix compounds may exhibit the absorption or emission of light in the same spectral regions as the fluorescein label. This situation does not arise often when testing food products, but it occurs, for example, for red wines [53]. The application of the proposed approach for such systems will require either an additional preprocessing of the samples to eliminate the interfering component(s) or change fluorescein to another fluorophore. The second factor is the structural differences in various aptamers, which could require different concentrations of stabilizing reagents, and the gain in the detection limit may vary. However, the choice of concentrations of the reaction media components is an integral part of the optimization for any aptamer-based analysis. For our systems, the choice will be only extended to additional reagents.

The possibility to implement the proposed assay as a “mix and detect” procedure, i.e., without any additional manipulations with the reaction mixture before the measurements determines its perspectives for onsite real-time use. The additional adaptation for this purpose will be a change in the used FA detection to available autonomous devices such as portable FA detectors or units for recording FA using serial communication devices [54,55].

Prospective tasks for further application of the proposed polycation-based enhancing approach is testing its combinations with other tools increasing anisotropy fluorescence of the bound tracer (such as the immobilization of the aptamer on additional anchoring carriers [4,16]) to possibly lower the detection limit further.

## 4. Conclusions

The aptamer-based fluorescence anisotropy assay proposed in this study realizes, for the first time, the application of polycations (poly-L-lysine and polyethylene glycol) and high concentrations of magnesium cations to modulate the rotation of the formed aptamer–tracer complexes and enhance fluorescence anisotropy signals in the competitive aptamer-based assay. This work adds a new tool for lowering the detection limit of the aptamer-based fluorescence anisotropy assay that differs from earlier considered modulators and can be implemented in a one-step technique. The application of this approach resulted in a 12-fold increased sensitivity of the AFB1 detection, reaching the levels of practical interest for screening control of agricultural products contamination.

## Figures and Tables

**Table 1:** The comparison of K_D_ values of variants of stem–loop anti-AFB1 aptamer with different lengths of the stem region.

Lines	Short Indications	Oligonucleotide Sequences	Stem Length	*** H-Bonds	K_D_ (nM)
1	Initial (untruncated)	*GTT* GGG CAC GTG TTG TCT CTC TGT GTC T * CG TGC CC*T TCG CTA GGC CCA CA* **	7	20	173.1 ± 9.5
2	22 nt	C GTG TTG TCT CTC TGT GTC TCG	2	6	2751 ± 443 ****
3	26 nt	CAC GTG TTG TCT CTC TGT GTC TCG TG	4	11	271.8 ± 51.1 ****
4	32 nt	GGG CAC GTG TTG TCT CTC TGT GTC TCG TGC CC	7	20	163.7 ± 19.0
5	38 nt	GTT GGG CAC GTG TTG TCT CTC TGT GTC TCG TGC CCA AC	10	27	123.0 ± 24.4
6	42 nt	GG GTT GGG CAC GTTG TTG TCT CTC TGT GTC TCG TGC CCA ACC C	12	33	218.0 ± 13.2
7	46 nt	T TGG GTT GGG CAC GTG TTG TCT CTC TGT GTC TCG TGC CCA ACC CAA	14	37	147.5 ± 14.5
8	54 nt	T TGG TTG G GTT GGG CAC GTG TTG TCT CTC TGT GTC TCG TGC CCA ACC CAA CCA A	18	47	559.7 ± 45.3

* loop region—highlighted in gray, ** tail region—*italicized and underscored*, *** H-bonds between nucleobases in stem region of aptamer, **** for these cases, the direct obtaining of saturation was unreachable; therefore, the K_D_ values were estimated, assuming that the FA value of completely bound AFB1-EDF is the same as for aptamer 32 nt.

**Table 2:** The recovery’s of AFB1 obtained using the proposed FA aptamer assay. The concentration values for spiked samples are indicated (n = 3).

Sample	Added (μg/kg)	Found (μg/kg)	Recovery (%)
Almond flour	200	242.1 ± 50.5	121.0
	100	121.2 ± 33.5	121.1
	75	91.6 ± 20.1	122.1
	30	33.8 ± 15.8	112.7
	10	12.3 ± 4.6	123.3

**Table 3:** Determination of AFB1 in almond samples by HPLC-MS/MS and the developed fluorescence anisotropy aptamer assay (n = 3).

Sample	Found AFB1 (μg/kg), HPLC-MS/MS Technique	Found AFB1 (μg/kg), FA-Based Assay	Recovery for FA-Based Assay (%)
1	376.3 ±12.6	416.1 ± 24.9	110.6 ± 6.0
2	282.2 ± 9.6	302.2 ± 9.1	107.1 ± 3.0
3	188.2 ± 4.5	212.9 ± 26.7	113.2 ± 12,5
4	112.9 ± 0.7	118.6 ± 19.3	105.0 ± 16.3
5	89.4 ± 0.3	75.0 ± 10.2	83.9 ± 13.7
6	37.8 ± 2.2	39.7 ± 10.4	105.1 ± 26.2
7	not detected	<LOD	-
8	not detected	<LOD	-

**Table 4:** Comparison of the analytical performance of fluorescence anisotropy systems for AFB1 detection.

Methods	Complexity	Linear (L)/Dynamic (D) Range (ng/mL)	LOD (ng/mL)	Tested Samples	Reference
Immunoassays					
Competition between native ligand and labeled ligand	One-step	-	5	Popcorn, corn, sorghum, peanut paste, peanut butter	[51]
	One-step	(L) 16.25–33.49	13.12	-	[28]
	One-step	(L) 3–84	1	beer	[27]
	One-step	(L) 92.76–252.32	20	tea leaves	[29]
	One-step	(L) 8.6–63.7	1	leaves of medicinal plants	[52]
Aptamer assays					
Polycation-assisted competitive aptamer assay with labeled ligand	One-step	(L) 4.0 ± 0.4–137.8 ± 8.0	0.72 ± 0.3	Almond flour	This work
Competitive aptamer assay with labeled ligand	One-step	(L) 15.0 ± 3.4–117.4 ± 11.2	9.7 ± 1.6	Almond flour	This work
Protein-encoded aptamer nanomachines and isothermal exponential amplification	Multi-step	-	0.000078	-	[50]
Direct labeling with guanine sensitive tetramethylrhodamine label	One-step	(D) 0.62–312.27	0.62	Serum; urine, wine, beer	[14]
Ligand-induced strand displacement with streptavidin anchor	Multi-step	(D) 0.08–39	0.019	White	[15]
Physical absorption of fluorescein-labeled aptamer on graphene oxide	Multi-step	(L) 0.015–1.56	0.015	Rice	[49]
Direct labeling with guanine sensitive tetramethylrhodamine label enhanced by strand displacement of guanine-rich ssDNA	One-step	(D) 0.04–9.74	0.039	Grape juice, milk, tap water	[31]
Ligand-induced strand displacement with IgG anchor	Multi-step	(D) 0.008–31.2	0.008	Grape juice, white wine, tap water	[30]

## Data Availability

The data presented in this study are available upon request from the corresponding author.

## Supplementary Materials

The following supporting information can be downloaded at: https://www.mdpi.com/article/10.3390/s24103230/s1, Figure S1: The dependencies of fluorescence (A), the change in fluorescence anisotropy (B), and the percentage of the bound fraction (C) of AFB1-EDF on the concentration of the untruncated stem–loop aptamer in different buffers: 1—20 mM Tris-Acetate, 100 mM Na Acetate, 10 mM MgAcetate2, 10 mM CaCl_2_, pH 8.4; 2—20 mM Tris- Acetate, 100 mM NaAcetate, pH 8.4; 3—20 mM Tris-Acetate, 20 mM MgAcetate2, pH 8.4; 4—20 mM Tris-Acetate, 100 mM NaAcetate, 20 mM MgAcetate2, pH 8.4 (n = 3); Table S1: The comparison of dissociation constants of the anti-AFB1 loop-stem aptamer in different buffer compositions; Figure S2: The dependencies of fluorescence (A), fluorescence anisotropy (B), and the percentage of bound fraction (C) of AFB1-EDF on the concentration of initial aptamer at different pH (n = 3); Table S2: The comparison of dissociation constants of the initial anti-AFB1 loop-stem aptamer at different pH; Figure S3: The dependencies of fluorescence (A) and change in fluorescence anisotropy (B) of AFB1-EDF in the presence of aptamer 38 nt (1) and its conjugates with streptavidin at ratio 2:1 (2) and 1:2 (3) (n = 3).
