# Supplementary material for: Polycations as Aptamer-Binding Modulators for Sensitive Fluorescence Anisotropy Assay of Aflatoxin B1
Source: Sensors (Basel). 2024 May 19;24(10):3230. doi: 10.3390/s24103230 (PMC11125339; doi:10.3390/s24103230)
Supplement: Supplementary file 1 [file sensors-24-03230-s001.zip › sensors-2953441-supplementary.pdf]

Supplementary Materials

Article

Polycations as aptamer-binding modulators for sensitive fluorescence anisotropy assay of aflatoxin B1

*Alexey V. Samokhvalov [1], Alena A. Mironova [1], Sergei A. Eremin [2], Anatoly V. Zherdev [1] and Boris B. Dzantiev [1]**

[1] *A.N. Bach Institute of Biochemistry, Research Center of Biotechnology, Russian Academy of Sciences, 119071, Moscow, Russia*

[2] *Faculty of Chemistry, M.V. Lomonosov Moscow State University, Moscow 119991, Russia*

*   *Correspondence: dzantiev@inbi.ras.r*

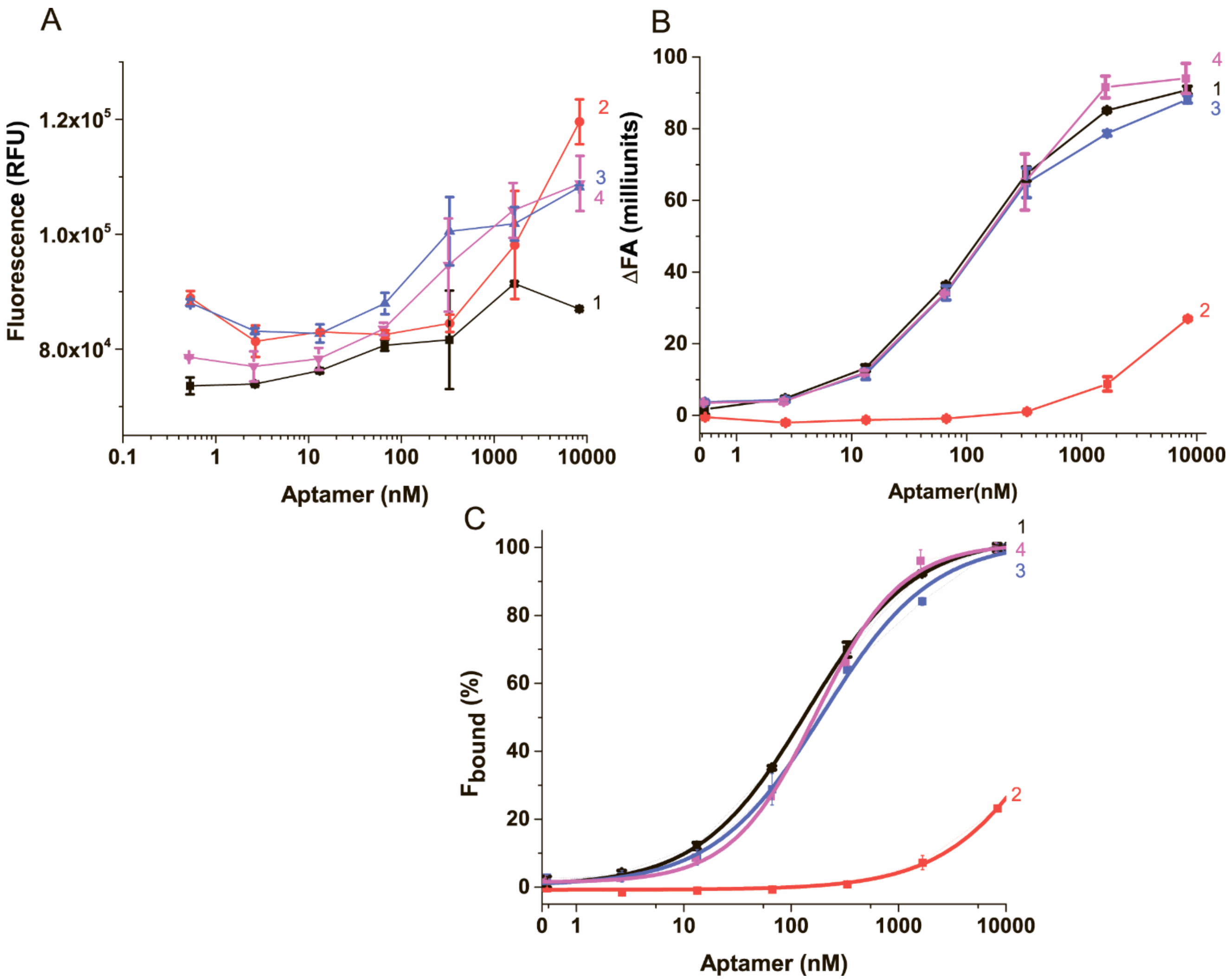

Figure S1. The dependencies of fluorescence (A), the change of fluorescence anisotropy (B), and the percentage of the bound fraction(C) of AFB_1-EDF on the concentration of the untruncated stem-loop aptamer in different buffers: 1 – 20 mM Tris-Acetate, 100 mM Na Acetate, 10 mM $MgAcetate_2$, 10 mM $CaCl_2$, pH 8.4; 2 – 20 mM Tris- Acetate, 100 mM NaAcetate, pH 8.4; 3 – 20 mM Tris- Acetate, 20 mM $MgAcetate_2$, pH 8.4; 4 – 20 mM Tris-Acetate, 100 mM NaAcetate, 20 mM $MgAcetate_2$, pH 8.4 (n=3)

Table S1. The comparison of dissociation constants of the anti-AFB1 loop-stem aptamer in different buffer compositions

#	Buffer composition	K_D (nM)
1	20 mM Tris-Acetate, 100 mM NaAcetate, 10 mM $MgAcetate_2$, 10 mM $CaCl_2$, pH 8.4	145.0±5.3
2	20 mM Tris-Acetate, 100 mM NaAcetate, pH 8.4	30500±4200*
3	20 mM Tris-Acetate, 20 mM $MgAcetate_2$, pH 8.4	190±28.1
4	20 mM Tris-Acetate, 100 mM NaAcetate, 20 mM $MgAcetate_2$, pH 8.4	173.1±9.5

*the K_D value was estimated with an assumption that maxFA is the same as for the buffer#1 composition

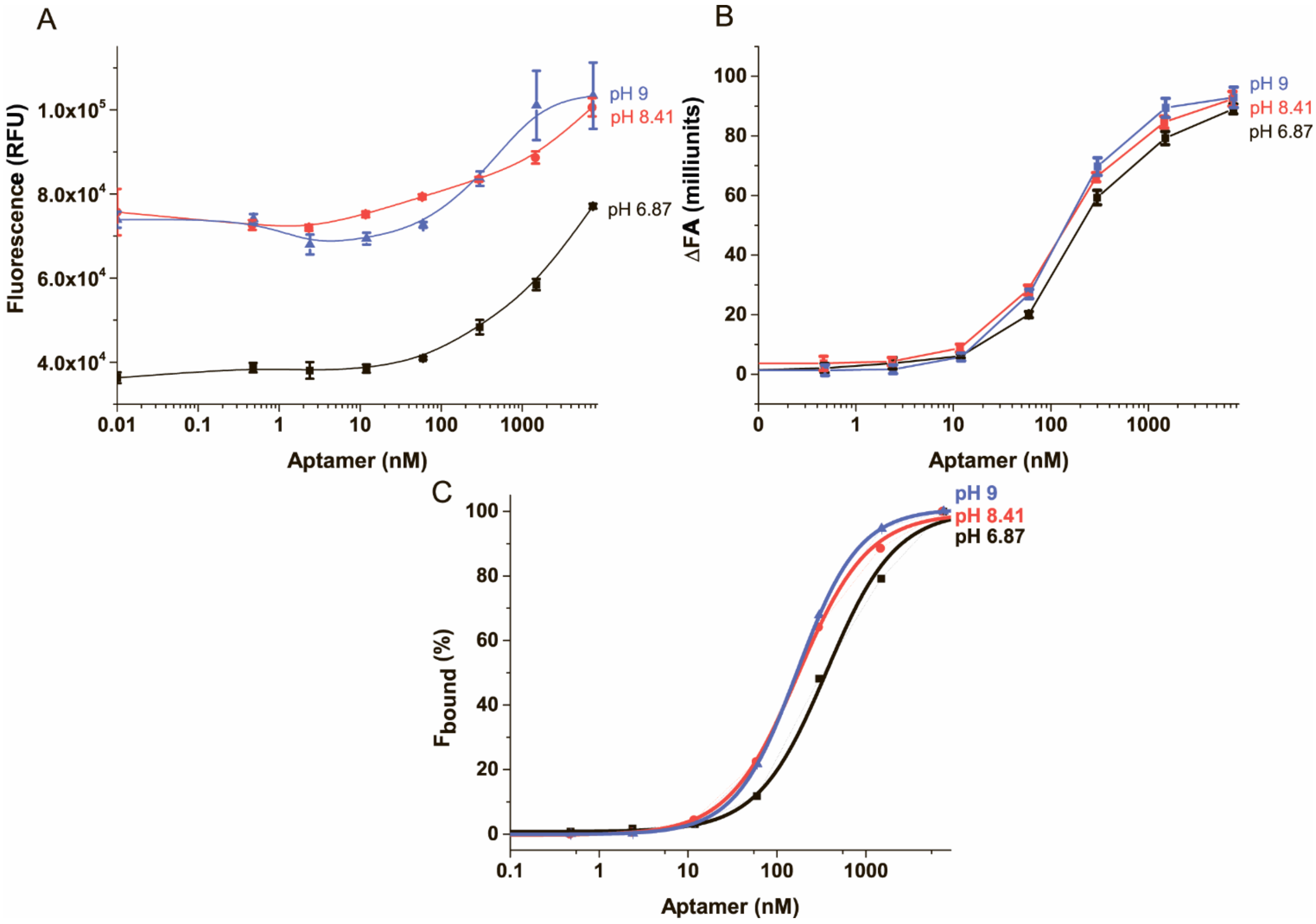

Figure S2. The dependencies of fluorescence (A), fluorescence anisotropy (B), and the percentage of bound fraction (C) of AFB1-EDF on the concentration of initial aptamer at different pH (n=3)

Table S2. The comparison of dissociation constants of the initial anti-AFB1 loop-stem aptamer at different pH

pH	Buffer composition	K_D (nM)
pH 6.8	20 mM Tris-Acetate, 100 mM NaAcetate, 20 mM $MgAcetate_2$	362.5±33.5
pH 8.4		173.1±9.5
pH 9		168.8±1.1

Synthesis of the aptamer-protein complexes

500 μL of 4 μM prepared 5'-biotinylated aptamer 38nt in TB was mixed with 500 μL of 4 or 8 μM streptavidin in TB. Then the obtained stock solutions were incubated for 1 h at room temperature with gentle stirring. After the series of consecutive 5-fold dilutions were prepared and mixed with an equal volume of AFB1-EDF (a final concentration of 5 nM). Each well contains 100μL of solution.

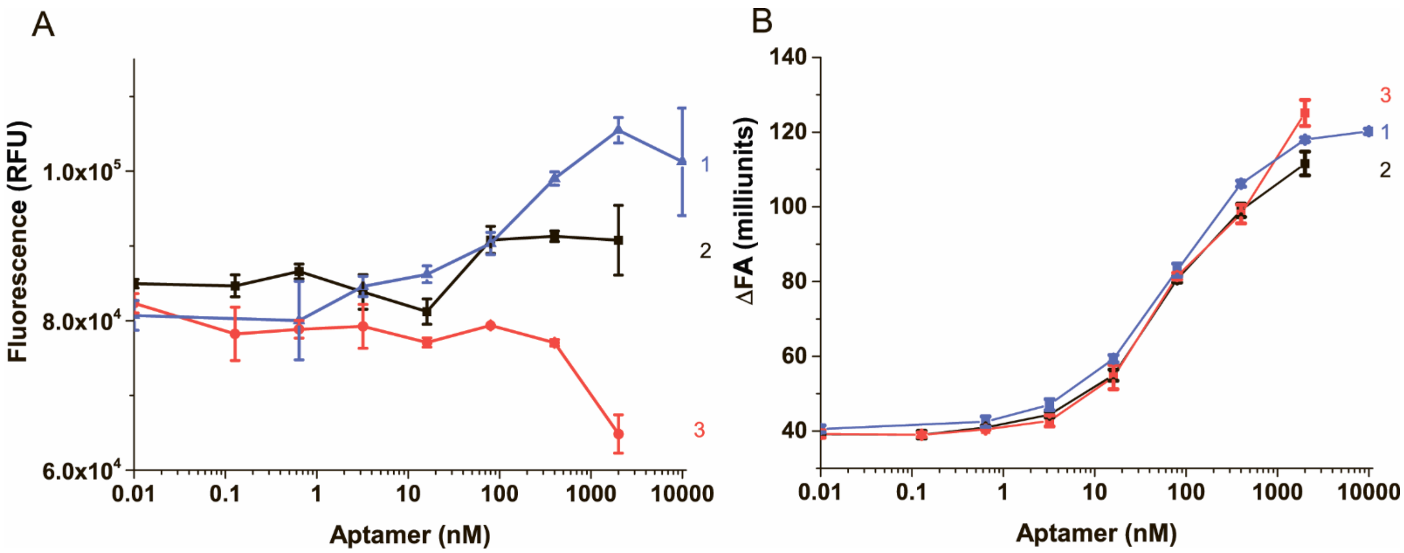

Figure S3. The dependencies of fluorescence (A) and change of fluorescence anisotropy (B) of AFB1-EDF in the presence of aptamer 38nt (1) and its conjugates with streptavidin at ratio 2:1 (2) and 1:2 (3). (n=3)
